# Expectation and variance of the estimator of the maximized selection response of linear selection indices with normal distribution

**DOI:** 10.1007/s00122-020-03629-6

**Published:** 2020-06-20

**Authors:** J. Jesus Cerón-Rojas, Jose Crossa

**Affiliations:** Biometrics and Statistics Unit, International Maize and Wheat Improvement Center (CIMMYT), Apdo. Postal 6-641, 06600 Mexico City, Mexico

## Abstract

**Key message:**

The expectation and variance of the estimator of the maximized index selection response allow the breeders to construct confidence intervals and to complete the analysis of a selection process.

**Abstract:**

The maximized selection response and the correlation of the linear selection index (LSI) with the net genetic merit are the main criterion to compare the efficiency of any LSI. The estimator of the maximized selection response is the square root of the variance of the estimated LSI values multiplied by the selection intensity. The expectation and variance of this estimator allow the breeder to construct confidence intervals and determine the appropriate sample size to complete the analysis of a selection process. Assuming that the estimated LSI values have normal distribution, we obtained those two parameters as follows. First, with the Fourier transform, we found the distribution of the variance of the estimated LSI values, which was a Gamma distribution; therefore, the expectation and variance of this distribution were the expectation and variance of the variance of the estimated LSI values. Second, with these results, we obtained the expectation and the variance of the estimator of the selection response using the Delta method. We validated the theoretical results in the phenotypic selection context using real and simulated dataset. With the simulated dataset, we compared the LSI efficiency when the genotypic covariance matrix is known *versus* when this matrix is estimated; the differences were not significant. We concluded that our results are valid for any LSI with normal distribution and that the method described in this work is useful for finding the expectation and variance of the estimator of any LSI response in the phenotypic or genomic selection context.

## Introduction

The maximized selection response and the correlation of the linear selection index (LSI) with the net genetic merit are the main criterion to compare the efficiency of any LSI. The selection response is the expectation of the net genetic merit of the selected individuals when the mean of the original population is zero, whereas the net genetic merit is a linear combination of the true unobservable breeding values of traits weighted by their respective economic values (Smith 1936; Cochran 1951). The LSI theory is divided into two main parts: (1) the unconstrained LSI (Smith 1936) and (2) the constrained LSI (Kempthorne and Nordskog 1959; Mallard 1972). The constrained LSI imposes restrictions on the expected genetic gain (or multitrait selection response) of some traits to make some of them change their expected genetic gain values based on a predetermined level, while the rest of them remain without restrictions. This index is the most general LSI, and it includes the unconstrained LSI as a particular case.

The unconstrained and constrained LSI can be a linear combination of phenotypic values (Smith 1936; Mallard 1972), genomic estimated breeding values (GEBV) (Ceron-Rojas et al. 2015; Cerón-Rojas and Crossa 2019), or phenotypic values and GEBV (Dekkers 2007) jointly. It can also be a linear combination of phenotypic values and marker scores (Lande and Thompson 1990). Thus, there are three main kinds of LSI: phenotypic, genomic and marker. The main advantage of the LSI based on GEBV over the other indices lies in the possibility of reducing the intervals between selection cycles by more than two-thirds.

The aims of any LSI are to predict the net genetic merit values of the candidates for selection, select parents for the next generation and maximize the selection response. When the phenotypic and genotypic variance and covariance are known, the maximized selection response is optimum and the LSI is the best linear predictor of the net genetic merit; in addition, the correlation between the net genetic merit and the LSI is maximized, and the mean prediction error is minimized.

The estimator of the maximized selection response is the square root of the variance of the estimated LSI values multiplied by the selection intensity. In this case, the phenotypic and genotypic variances and covariance are estimated and the expectation and variance of the estimator of the maximized selection response are unknown. Then, methods to find the expectation and variance of the estimator of the maximized LSI selection response are of interest to the breeder because they are important to complete the analysis of a selection process and because they allow the breeder to construct confidence intervals and determine the appropriate sample size for each selection cycle in a selection program.

The unconstrained and constrained linear phenotypic selection index (LPSI and CLPSI, respectively) theory was developed under the assumptions that the genotypic values that make up the net genetic merit are composed entirely of the additive effects of genes and that the LPSI (CLPSI) and the net genetic merit have bivariate normal distribution (Smith 1936, Kempthorne and Nordkog 1959; Mallard 1972). The major advantage of these indices is that they assign higher weights to traits whose differences are genetic. Their disadvantages are that they require large amounts of information, economic weights are difficult to assign and the sampling error could be large. Ceron-Rojas et al. (2015) and Cerón-Rojas and Crossa (2019) extended the LPSI and CLPSI theory to the genomic selection context and developed an unconstrained and a constrained linear genomic selection index (LGSI and CLGSI, respectively).

In the LPSI context, Tallis (1960) derived a large sample variance of LPSI weights for individually selecting any number of traits and the estimated LPSI selection response when phenotypic and genetic parameters are estimated in a half-sib analysis; however, the expressions are complicated and do not allow identifying situations where selection indices are likely to be inefficient. Williams (1962a) obtained an exact formula for the sampling variance of the index weights but for only two traits of a specific experimental design. Harris (1964) utilized the Delta method to determine the sampling properties of the index; however, the results are confusing and the author did not present a simple and general formula to find the expectation and variance of the estimator of the LPSI selection response. Hayes and Hill (1980) proposed a transformation of the trait variables used for constructing genetic selection indices, such that the sampling properties of the LPSI weights can be easily computed using a general formula; however, the formula depends on the transformation of the trait variables, which negatively affects the estimated LPSI selection response.

Assuming that the estimated LPSI and CLPSI values have normal distribution (we corroborated the normality assumption using graphical methods and normality tests), we present a simple and general formula to find the expectation and variance of the estimator of the maximized LPSI and CLPSI selection response, which we obtained in two steps. First, we obtained the distribution of the variance of the estimated LPSI and CLPSI values using the Fourier transform (Springer 1979, Chapters 2 and 9). Their distribution was a Gamma distribution, and therefore, the expectation and variance of this distribution were the expectation and variance of the variance of the estimated LPSI and CLPSI values.

In the second step, using the results obtained in the first step, we found the expectation and the variance of the estimator of the maximized LPSI and CLPSI selection responses using the Delta method. We validated the theoretical results using real and simulated dataset. In addition, with the simulated dataset, we compared the LPSI and CLPSI parameters when the genotypic covariance matrix is known *versus* when this matrix is estimated by restricted maximum likelihood (REML). We did this because while the sampling properties of the estimator of the phenotypic covariance matrix are well known (Rencher and Schaalje 2008), the sampling properties of the estimator of the genotypic covariance matrix are not well known. The results indicated that the differences are not significant. We concluded that our method is useful to find the expectation and variance of the estimator of the maximized selection response for any LSI with normal distribution.

## Materials and methods

### The net genetic merit and the LPSI

The individual net genetic merit is

1 $$H = {\mathbf{w^{\prime}g}},$$

where $${\mathbf{g^{\prime}}} = [\begin{array}{*{20}c} {g_{1} } & {g_{2} } & {...} & {g_{t} } \\ \end{array} ]$$ and $${\mathbf{w^{\prime}}} = [\begin{array}{*{20}c} {w_{1} } & {w_{2} } & {...} & {w_{t} } \\ \end{array} ]$$ ($t =$ number of traits) are vectors of true unobservable breeding values and known economic values, respectively. The individual linear phenotypic selection index (LPSI) is

2 $$I = {\mathbf{b^{\prime}y}},$$

where $${\mathbf{b^{\prime}}} = [\begin{array}{*{20}c} {b_{1} } & {b_{2} } & {...} & {b_{t} } \\ \end{array} ]$$ is the LPSI vector of coefficients, and $${\mathbf{y^{\prime}}} = [\begin{array}{*{20}c} {y_{1} } & {y_{2} } & {...} & {y_{t} } \\ \end{array} ]$$ is the vector of the traits of interest. The variances of $H$ and $I$ are $\sigma_{H}^{2} = {\mathbf{w^{\prime}Cw}}$ and $\sigma_{I}^{2} = {\mathbf{b^{\prime}Pb}}$, respectively, where ${\mathbf{C}}$ and ${\mathbf{P}}$ are $t \times t$ covariance matrices of genotypic (**g**) and trait phenotypic values (${\mathbf{y}}$), respectively.

### The LPSI selection response

The LPSI selection response ($R$) is the expectation of $H$ (Eq. 1) for a proportion $p$ of individuals selected and can be written as

3 $$R = k\sigma_{H} \rho_{HI} ,$$

where $k = \frac{z(u)}{p}$ is the intensity of selection, $z(u) = \frac{{\exp \{ - 0.5u^{2} \} }}{{\sqrt {2\pi } }}$ is the height of the ordinate of the normal curve and $u = \frac{{I - \mu_{I} }}{{\sigma_{I} }}$ is the truncation point, whereas $\mu_{I}$ and $\sigma_{I} = \sqrt {{\mathbf{b^{\prime}Pb}}}$ are the mean and standard deviations of the variance of $I$ (Eq. 2); $\sigma_{H} = \sqrt {{\mathbf{w^{\prime}Cw}}}$ is the standard deviation of the variance $H$ and $\rho = \frac{{{\mathbf{w^{\prime}Cb}}}}{{\sqrt {{\mathbf{w^{\prime}Cw}}} \sqrt {{\mathbf{b^{\prime}Pb}}} }}$ is the correlation between $H$ and the LPSI, whereas $\sigma_{HI} = {\mathbf{w^{\prime}Cb}}$ is the covariance between $H$ and $I$.

The genetic gain in Eq. (3) will be larger as $p$ becomes smaller—i.e., as the selection intensity becomes more intense. Equation (3) is the same for all LSI; the only change is the type of information (phenotypic or genomic) and restrictions used when the index vector of coefficients is obtained to predict $H$ and to maximize Eq. (3).

### The maximized LPSI selection response and coefficient of correlation

The maximized LPSI selection response and the correlation of the LPSI with the net genetic merit are

4a $$R_{\max } = k\sqrt {{\mathbf{b^{\prime}Pb}}} = k\sigma_{I} ,$$

4b $$\rho_{{\max}} = \frac{{\sqrt {{\mathbf{b^{\prime}Pb}}} }}{{\sqrt {{\mathbf{w^{\prime}Cw}}} }},$$

respectively, where ${\mathbf{b}} = {\mathbf{P}}^{ - 1} {\mathbf{Cw}}$ (Cerón-Rojas and Crossa 2018, Chapter 2). Equation (4a) predicts the mean improvement in $H$ due to indirect selection on $I$ and is proportional to the standard deviation of the LPSI variance ($\sigma_{I}$) and the selection intensity $k$. Whereas in Eq. (3) $R$ can take any value, in Eq. (4a) $R_{\max }$ gives the maximum value of Eq. (3). This is the main difference between the two equations.

### The expected genetic gain per trait

The main objective of the CLPSI is to maximize Eq. (3) under some restrictions imposed on the expected genetic gain per trait (${\mathbf{E}}$), which can be written as

5 $${\mathbf{E}} = k\frac{{{\mathbf{Cb}}}}{{\sqrt {{\mathbf{b^{\prime}Pb}}} }}.$$

We defined all the terms in Eq. (5) earlier. The type of restriction imposed on Eq. (5) can be a null restriction (RLPSI) or a predetermined constraint (CLPSI). Thus, let $${\mathbf{d^{\prime}}} = [\begin{array}{*{20}c} {d_{1} } & {d_{2} } & \cdots & {d_{r} } \\ \end{array} ]$$ be a vector of $r$ constraints and assume that $\mu_{q}$ is the population mean of the *q*^th^ trait ($q = 1,2, \cdots ,r$, and $r$ is the number of constraints) before selection. The CLPSI changes $\mu_{q}$ to $\mu_{q} + d_{q}$, where $d_{q}$ is a predetermined change in $\mu_{q}$ imposed by the breeder. When ${\mathbf{d}}$ is a null vector, we have a null restricted LPSI (RLPSI), which is a particular case of the CLPSI. The restriction effects will be observed on the CLPSI expected genetic gains per trait (Eq. 5), where each restricted trait will have an expected genetic gain according to the $${\mathbf{d^{\prime}}} = [\begin{array}{*{20}c} {d_{1} } & {d_{2} } & \cdots & {d_{r} } \\ \end{array} ]$$ values imposed by the breeder.

Equation (5) is the same for all LSI; the only change is the type of information (phenotypic or genomic) and restrictions used when the LSI vectors of coefficients are obtained to predict $H$ and to maximize Eq. (3).

### The CLPSI vector of coefficients

Let $${\mathbf{D}}^{^{\prime}}=\left[\begin{array}{ccc}\begin{array}{c}{d}_{r}\\ 0\\ \begin{array}{c}\vdots \\ 0\end{array}\end{array}& \begin{array}{c}0\\ {d}_{r}\\ \begin{array}{c}\vdots \\ 0\end{array}\end{array}& \begin{array}{ccc}\begin{array}{c}\dots \\ \dots \\ \begin{array}{c}\ddots \\ \dots \end{array}\end{array}& \begin{array}{c}0\\ 0\\ \begin{array}{c}\vdots \\ {d}_{r}\end{array}\end{array}& \begin{array}{c}{-d}_{1}\\ {-d}_{2}\\ \begin{array}{c}\vdots \\ {-d}_{r-1}\end{array}\end{array}\end{array}\end{array}\right]$$ be a Mallard (1972) matrix $(r - 1) \times r$ of predetermined proportional gains, where $d_{q}$ ($q = {1,2} \ldots {,}r$) is the $q^{th}$ element of vector $${\mathbf{d^{\prime}}} = [\begin{array}{*{20}c} {d_{1} } & {d_{2} } & \cdots & {d_{r} } \\ \end{array} ]$$, and let ${\mathbf{U^{\prime}}}$ be a matrix of 1′s and 0′s, where 1 indicates that the traits are restricted and 0 that the traits are not restricted (Kempthorne and Nordskog 1959). To obtain the CLPSI vector of coefficients, we minimized the mean-squared difference between $I$ and $H$, $E[(H - I)^{2} ]$, with respect to ${\mathbf{b}}$ under the restriction ${\mathbf{D^{\prime}U^{\prime}Cb}} = {\mathbf{0}}$, where ${\mathbf{C}}$ is the covariance matrix of genotypic values.

The CLPSI vector of coefficients is

6 $${{\varvec{\upbeta}}} = {\mathbf{Kb}},$$

where ${\mathbf{K}} = [{\mathbf{I}}_{t} - {\mathbf{Q}}]$, ${\mathbf{Q}} = {\mathbf{P}}^{ - 1} {\mathbf{M}}({\mathbf{M^{\prime}P}}^{ - 1} {\mathbf{M}})^{ - 1} {\mathbf{M^{\prime}}}$, ${\mathbf{M^{\prime}}} = {\mathbf{D^{\prime}U^{\prime}C}}$, ${\mathbf{I}}_{t}$ is an identity matrix of size $t \times t$ and ${\mathbf{b}} = {\mathbf{P}}^{ - 1} {\mathbf{Cw}}$. When ${\mathbf{d}}$ is a null vector, ${\mathbf{D}} = {\mathbf{U}}$, ${\mathbf{Q}} = {\mathbf{P}}^{ - 1} {\mathbf{CU}}({\mathbf{U^{\prime}CP}}^{ - 1} {\mathbf{CU}})^{ - 1} {\mathbf{U^{\prime}C}}$, and the CLPSI is the RLPSI. When ${\mathbf{D}} = {\mathbf{U}}$ and ${\mathbf{U^{\prime}}}$ is a null matrix, ${{\varvec{\upbeta}}} = {\mathbf{b}}$. Thus, the CLPSI is the most general linear phenotypic selection index and includes the LPSI and the RLPSI as particular cases.

### The maximized CLPSI selection response and coefficient of correlation

The maximized CLPSI selection response and the correlation of the LPSI with the net genetic merit are

7a $$R_{\max C} = k\sqrt {{\mathbf{\beta^{\prime}P\beta }}} = k\sigma_{{I_{C} }} ,$$

7b $$\rho_{\max C} = \frac{{\sqrt {{\mathbf{\beta^{\prime}P\beta }}} }}{{\sqrt {{\mathbf{w^{\prime}Cw}}} }},$$

respectively, where $k$ is the selection intensity. Under $r$ restrictions, Eq. (7a) predicts the mean improvement in $H$ due to indirect selection on $I_{C} = {\mathbf{\beta^{\prime}y}}$.

### Estimators of the LPSI and CLPSI vector of coefficients

We denote the restricted maximum likelihood (REML) estimators of matrices ${\mathbf{C}}$ and ${\mathbf{P}}$ as ${\hat{\mathbf{C}}}$ and ${\hat{\mathbf{P}}}$, respectively (Cerón-Rojas and Crossa 2018, Chapter 2), from where the LPSI and CLPSI vectors of coefficients (${\mathbf{b}} = {\mathbf{P}}^{ - 1} {\mathbf{Cw}}$ and ${{\varvec{\upbeta}}} = {\mathbf{Kb}}$) can be estimated, respectively, as

8 $${\hat{\mathbf{b}}} = {\hat{\mathbf{P}}}^{ - 1} {\hat{\mathbf{C}}\mathbf{w}}\quad {\text{and}}\quad {\hat{\mathbf{\beta }}} = {\mathbf{\hat{K}\hat{b}}},$$

where $${\hat{\mathbf{K}}} = [{\mathbf{I}}_{t} - {\hat{\mathbf{Q}}}]$$, $${\hat{\mathbf{Q}}} = {\hat{\mathbf{P}}}^{ - 1} {\hat{\mathbf{M}}}({\mathbf{\hat{M^{\prime}}\hat{P}}}^{ - 1} {\hat{\mathbf{M}}})^{ - 1} {\mathbf{\hat{M^{\prime}}}}$$ and $${\mathbf{\hat{M^{\prime}}}} = {\mathbf{D^{\prime}U^{\prime}\hat{C}}}$$.

### Estimators of LPSI and CLPSI

**Fig. 1:**
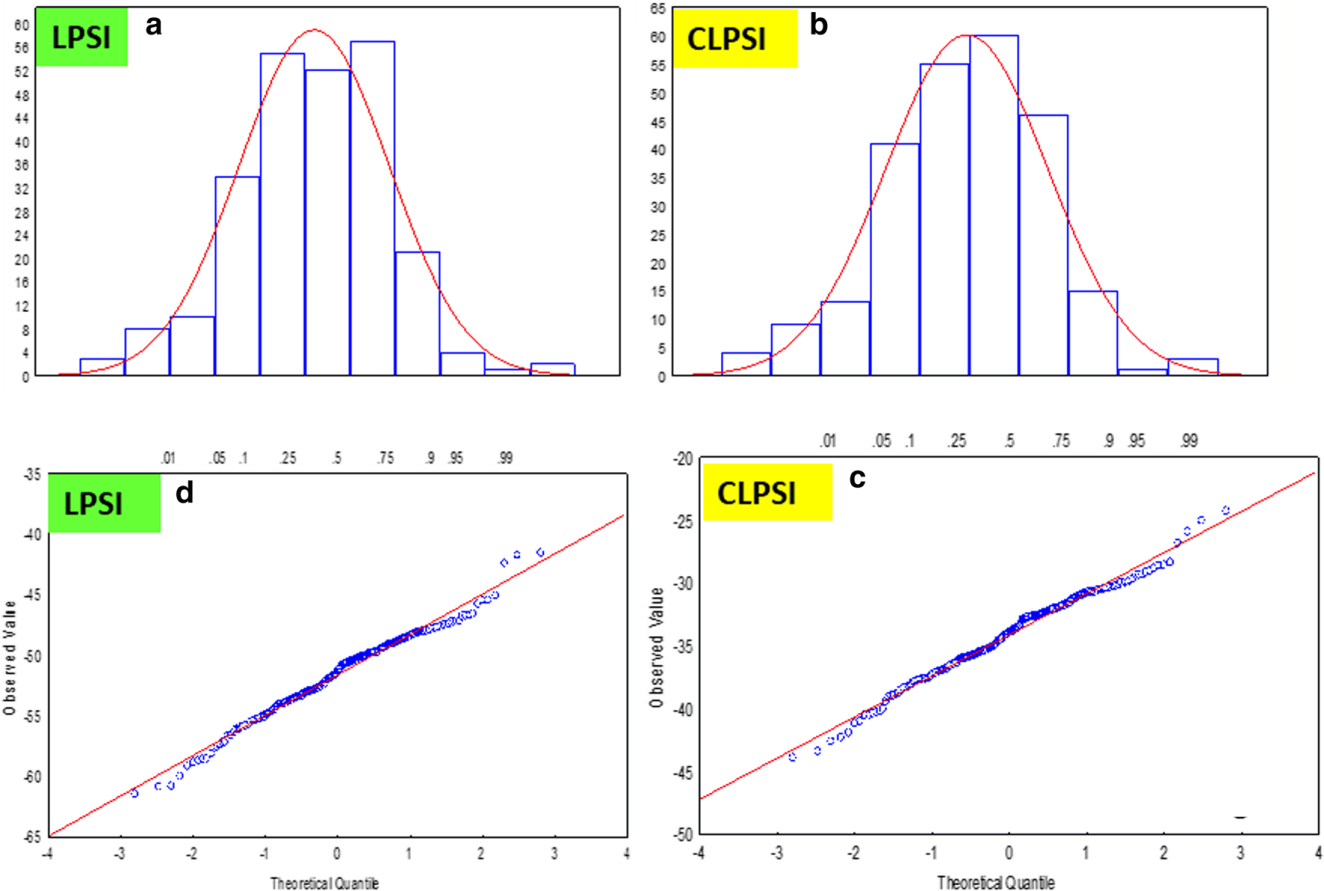
Histograms and quantile–quantile plots of the estimated LPSI (Fig. 1a, d, respectively) and CLPSI (Fig. 1b, c, respectively) values for a real dataset with four traits and 247 genotypes

By Eq. (8), the estimators of LPSI ($I = {\mathbf{b^{\prime}y}}$) and CLPSI ($I_{C} = {\mathbf{\beta^{\prime}y}}$) are

9 $$\hat{I} = {\mathbf{\hat{b^{\prime}}y}}\quad {\text{and}}\quad \hat{I}_{C} = {\mathbf{\hat{\beta^{\prime}}y}},$$

respectively. The $\hat{I}$ and $\hat{I}_{C}$ values (Eq. 9) are used to rank and select genotypes in the population. In this work, we assumed that the $\hat{I}$ and $\hat{I}_{C}$ values have normal distributions (Fig. 1).

### Estimator of the LPSI and CLPSI variances

The estimator of the variance of the LPSI ($\sigma_{I}^{2} = {\mathbf{b^{\prime}Pb}}$) is

10 $$S_{I}^{2} = \frac{1}{n - 1}\sum\limits_{j = 1}^{n} {(\hat{I}_{j} - \hat{m})^{2} } ,$$

where $\hat{m} = \frac{1}{n}\sum\nolimits_{j = 1}^{n} {\hat{I}_{j} }$ is the arithmetic means of the $\hat{I}$ values. In a similar manner, the estimator of the variance of the CLPSI ($\sigma_{{I_{C} }}^{2} = {\mathbf{\beta^{\prime}P\beta }}$) is

11 $$S_{{I_{C} }}^{2} = \frac{1}{n - 1}\sum\limits_{j = 1}^{n} {(\hat{I}_{{C_{j} }} - \hat{\mu })^{2} } ,$$

where $\hat{\mu } = \frac{1}{n}\sum\nolimits_{j = 1}^{n} {\hat{I}_{{C_{j} }} }$ is the arithmetic means of the $\hat{I}_{C}$ values. In both equations, $n$ is the size of the population in each selection cycle.

It is possible to estimate $\sigma_{I}^{2} = {\mathbf{b^{\prime}Pb}}$ as $\hat{\sigma }_{I}^{2} = {\mathbf{\hat{b^{\prime}}\hat{P}\hat{b}}}$, and $\sigma_{{I_{C} }}^{2} = {\mathbf{\beta^{\prime}P\beta }}$ as $\hat{\sigma }_{{I_{C} }}^{2} = {\mathbf{\hat{\beta^{\prime}}\hat{P}\hat{\beta }}}$; however, in this work, we found that the estimated values of $\hat{\sigma }_{I}^{2}$ and $\hat{\sigma }_{{I_{C} }}^{2}$ are the same as those of Eqs. (10) and (11), respectively. We estimated $\sigma_{I}^{2} = {\mathbf{b^{\prime}Pb}}$ and $\sigma_{{I_{C} }}^{2} = {\mathbf{\beta^{\prime}P\beta }}$ with $S_{I}^{2}$ and $S_{{I_{C} }}^{2}$, respectively, because when $\hat{I}$ and $\hat{I}_{C}$ have normal distribution, it is easier to find the distribution of the $S_{I}^{2}$ and $S_{{I_{C} }}^{2}$ values (Appendices A–D) than the distribution of the $\hat{\sigma }_{I}^{2} = {\mathbf{\hat{b^{\prime}}\hat{P}\hat{b}}}$ and $\hat{\sigma }_{{I_{C} }}^{2} = {\mathbf{\hat{\beta^{\prime}}\hat{P}\hat{\beta }}}$ values. The expectation and variance of $S_{I}^{2}$ and $S_{{I_{C} }}^{2}$ are useful to find the expectation and variance of the estimator of the maximized selection responses of both indices.

## Estimators of the maximized selection responses

By Eqs. (10) and (11), the estimators of the maximized LPSI and CLPSI selection responses are

12 $$\hat{R}_{\max } = k\sqrt {S_{I}^{2} }$$

and

13 $$\hat{R}_{\max C} = k\sqrt {S_{{I_{C} }}^{2} } ,$$

respectively.

### Testing the normality assumption to the estimated LPSI and CLPSI values

For the real dataset, we corroborated the normality assumption to the estimated LPSI and CLPSI values using graphical methods (histograms and normal quantile–quantile plots) and analytical test procedures (the Shapiro–Wilk and Kolmogorov–Smirnov normality tests), while for the simulated dataset, we used only analytical test procedures.

If the estimated LPSI and CLPSI values have normal distribution, the histograms of the values of both indices should not show a strong negative or positive skew in the LPSI and CLPSI values seen in the histogram (Fig. 1a, b). In a similar manner, if the estimated LPSI and CLPSI values are normally distributed, the LPSI and CLPSI values should form a straight line in the quantile–quantile plots (Fig. 1c, d). If there are departures from normality, the LPSI and CLPSI values should show up as various kinds of nonlinearity, e.g., S-shaped or banana-shaped in the quantile–quantile plots (Crawley 2015).

We tested the null hypothesis that the estimated LPSI and CLPSI values have normal distribution using the Shapiro–Wilk and Kolmogorov–Smirnov normality tests. The statistical value of the Shapiro–Wilk test should be close to 1.0 to accept the null hypothesis, while the statistical value of the Kolmogorov–Smirnov test should be close to 0.0 to accept the null hypothesis (Crawley 2015).

### Estimator of the maximized LPSI and CLPSI selection responses using ${\mathbf{C}}$*versus*${\hat{\mathbf{C}}}$.

Based on the Cauchy–Schwarz inequality, in Appendix A (Eqs. A1–A3), we describe an upper boundary for the maximized LPSI and CLPSI selection responses. By Eq. (A2), $k\sqrt {{\mathbf{w^{\prime}Cw}}}$ is the maximum possible value of the maximized LPSI selection response ($R_{\max } = k\sqrt {{\mathbf{b^{\prime}Pb}}} = k\sqrt {{\mathbf{w^{\prime}CP}}^{ - 1} {\mathbf{Cw}}}$); i.e., $R_{\max } \le k\sqrt {{\mathbf{w^{\prime}Cw}}}$. In a similar manner, by Eq. (A3), $k\sqrt {{\mathbf{\delta^{\prime}C\delta }}}$ is the maximum possible value of the maximized CLPSI selection response ($R_{\max C} = k\sqrt {{\mathbf{\beta^{\prime}P\beta }}}$), i.e., $R_{\max C} \le k\sqrt {{\mathbf{\delta^{\prime}C\delta }}}$.

In the simulated datasets, the true genotypic covariance matrix ${\mathbf{C}}$ is known. Thus, in this case, it is possible to estimate the LPSI vector of coefficients as ${\hat{\mathbf{b}}} = {\hat{\mathbf{P}}}^{ - 1} {\hat{\mathbf{C}}\mathbf{w}}$, where ${\hat{\mathbf{C}}}$ is the REML of ${\mathbf{C}}$, and as ${\tilde{\mathbf{b}}} = {\hat{\mathbf{P}}}^{ - 1} {\mathbf{Cw}}$, where ${\mathbf{C}}$ is known. In the CLPSI context, we would have ${\hat{\mathbf{\beta }}} = {\mathbf{\hat{K}\hat{b}}}$ (Eq. 8) and ${\tilde{\mathbf{\beta }}} = {\mathbf{\tilde{K}\tilde{b}}}$, where ${\tilde{\mathbf{K}}} = [{\mathbf{I}}_{t} - {\tilde{\mathbf{Q}}}]$, ${\tilde{\mathbf{Q}}} = {\hat{\mathbf{P}}}^{ - 1} {\tilde{\mathbf{M}}}({\mathbf{\tilde{M^{\prime}}\hat{P}}}^{ - 1} {\tilde{\mathbf{M}}})^{ - 1} {\mathbf{\tilde{M^{\prime}}}}$ and ${\mathbf{\tilde{M^{\prime}}}} = {\mathbf{D^{\prime}U^{\prime}C}}$. In both cases, the only difference among the estimator of the indices vectors of coefficients is matrix ${\mathbf{C}}$. With these results, we can compare the maximized LPSI selection response when this is estimated as $\hat{R}_{\max } = k\sqrt {{\mathbf{w^{\prime}\hat{C}\hat{P}}}^{ - 1} {\hat{\mathbf{C}}\mathbf{w}}}$ and as $\tilde{R}_{\max } = k\sqrt {{\mathbf{w^{\prime}C\hat{P}}}^{ - 1} {\mathbf{Cw}}}$, where the only difference is matrices ${\hat{\mathbf{C}}}$ and ${\mathbf{C}}$. If ${\hat{\mathbf{C}}}$ is a good estimate of ${\mathbf{C}}$, we would expect that $\hat{R}_{\max }$ and $\tilde{R}_{\max }$ be equivalent, and we would assume that ${\hat{\mathbf{C}}}$ is a good estimator of ${\mathbf{C}}$. The same is true for the CLPSI.

### Variance and confidence interval for the LPSI and CLPSI correlation coefficients using ${\mathbf{C}}$ and ${\hat{\mathbf{C}}}$.

In Appendix A (Eqs. A4 and A5), we describe the standard deviation of the variance of $\rho_{\max }$ (Eq. 4b) and $\rho_{\max C}$(Eq. 7b) and one form to construct an approximated 100(1 − $\alpha$)% confidence interval for $\rho_{\max }$ and $\rho_{\max C}$. In the simulated dataset selection context, for the REML estimate ${\hat{\mathbf{C}}}$, the estimated LPSI and CLPSI correlation coefficients ($\rho_{\max }$ and $\rho_{\max C}$, respectively) are $\hat{r}_{\max } = \frac{{\sqrt {{\mathbf{\hat{b^{\prime}}\hat{P}\hat{b}}}} }}{{\sqrt {{\mathbf{w^{\prime}\hat{C}w}}} }}$ and $\hat{r}_{\max C} = \frac{{\sqrt {{\mathbf{\hat{\beta }^{\prime}\hat{P}\hat{\beta }}}} }}{{\sqrt {{\mathbf{w^{\prime}\hat{C}w}}} }}$, respectively, whereas for matrix ${\mathbf{C}}$, those estimates are $\tilde{\rho }_{\max } = \frac{{\sqrt {{\mathbf{\tilde{b^{\prime}}\hat{P}\tilde{b}}}} }}{{\sqrt {{\mathbf{w^{\prime}Cw}}} }}$ and $\tilde{\rho }_{\max C} = \frac{{\sqrt {{\mathbf{\tilde{\beta }^{\prime}\hat{P}\tilde{\beta }}}} }}{{\sqrt {{\mathbf{w^{\prime}Cw}}} }}$, respectively, where ${\tilde{\mathbf{b}}} = {\hat{\mathbf{P}}}^{ - 1} {\mathbf{Cw}}$ and ${\tilde{\mathbf{\beta }}} = {\mathbf{\tilde{K}\tilde{b}}}$. The only difference of those estimates is matrices ${\hat{\mathbf{C}}}$ and ${\mathbf{C}}$. If ${\hat{\mathbf{C}}}$ is a good estimate of ${\mathbf{C}}$, we would expect that $\hat{r}_{\max }$ and $\tilde{\rho }_{\max }$, and $\hat{r}_{\max C}$ and $\tilde{\rho }_{\max C}$, be equivalent. In such a case, we would assume that ${\hat{\mathbf{C}}}$ is a good estimator of ${\mathbf{C}}$. Therefore, we compared these parameters in a similar manner as we did for the estimators of the maximized LPSI and CLPSI selection responses in the last subsection.

### Real data

To validate the theoretical results of the expectation and variance of the estimator of the maximized LPSI and CLPSI selection response, we used a real maize (*Zea mays* L.) F2 population with 247 genotypes and four phenotypic traits: grain yield (GY, t/ha), plant height (PHT, cm), ear height (EHT, cm) and anthesis days (AD, d), where we assumed that the breeding objective was to increase GY while decreasing PHT, EHT and AD. The vector of economic weights for GY, PHT, EHT and AD was $${\mathbf{w}}^{^{\prime}}=[\begin{array}{ccc}5& - 0.3& \begin{array}{cc}- 0.3& - 1\end{array}\end{array}]$$ for both indices. Beyene et al. (2015) described this dataset and denoted it as JMpop1 DTMA Mexico optimum environment.

We estimated ${\mathbf{P}}$ and ${\mathbf{C}}$ by REML, and we denoted such estimates as ${\hat{\mathbf{P}}}$ and ${\hat{\mathbf{C}}}$, i.e.,

$${\hat{\mathbf{P}}} = \left[ {\begin{array}{*{20}c} {1.40} & {4.69} & {3.25} & {0.12} \\ {4.69} & {130.57} & {68.39} & {0.80} \\ {3.25} & {68.39} & {68.22} & { - 0.72} \\ {0.12} & {0.80} & { - 0.72} & {1.44} \\ \end{array} } \right]$$ and $${\hat{\mathbf{C}}} = \left[ {\begin{array}{*{20}c} {0.94} & {3.76} & {2.62} & {0.29} \\ {3.76} & {72.24} & {43.81} & {1.99} \\ {2.61} & {43.81} & {35.60} & {0.31} \\ {0.29} & {1.99} & {0.31} & {0.90} \\ \end{array} } \right]$$. For illustration purposes only, in the CLPSI context, we restricted traits GY, PHT and EHT with vector $${\mathbf{d^{\prime}}} = [\begin{array}{*{20}c} {0.5} & { - 1.0} & { - 0.5} \\ \end{array} ]$$ and matrices $${\mathbf{U^{\prime}}} = \left[ {\begin{array}{*{20}c} 1 & 0 & 0 & 0 \\ 0 & 1 & 0 & 0 \\ 0 & 0 & 1 & 0 \\ \end{array} } \right]$$ and $${\mathbf{D^{\prime}}} = \left[ {\begin{array}{*{20}c} { - 0.5} & 0 & { - 0.5} \\ 0 & { - 0.5} & {1.0} \\ \end{array} } \right]$$, when we made selection. For both indices, the total proportion of retained value for this dataset was $p =$ 0.10 ($k = 1.755$).

### Simulated datasets

The datasets were simulated by Ceron-Rojas et al. (2015) with QU-GENE software (Podlich and Cooper 1998) using 2500 molecular markers and 315 quantitative trait loci (QTLs) for eight phenotypic selection cycles (C0 to C7), each with four traits ($T_{1}$, $T_{2}$, $T_{3}$ and $T_{4}$), 500 genotypes and four replicates for each genotype. The authors distributed the markers uniformly across ten chromosomes and the QTLs randomly across the ten chromosomes to simulate maize (*Zea mays* L.) populations. A different number of QTLs affected each of the four traits: 300, 100, 60, and 40, respectively. The common QTLs affecting the traits generated genotypic correlations of − 0.5, 0.4, 0.3, − 0.3, − 0.2, and 0.1 between $T_{1}$ and $T_{2}$, $T_{1}$ and $T_{3}$, $T_{1}$ and $T_{4}$, $T_{2}$ and $T_{3}$, $T_{2}$ and $T_{4}$, $T_{3}$ and $T_{4}$, respectively. The economic weights for $T_{1}$, $T_{2}$, $T_{3}$ and $T_{4}$ were 1, − 1, 1 and 1, respectively.

We used seven phenotypic selection cycles (C1 to C7) with $p =$ 0.10 ($k = 1.755$) in each cycle. We selected all four traits in each selection cycle. For illustration purposes only, in the CLPSI context, we restricted traits $T_{1}$, $T_{2}$ and $T_{3}$ with vector $${\mathbf{d^{\prime}}} = [\begin{array}{*{20}c} 5 & { - \,2} & 3 \\ \end{array} ]$$ and matrices $${\mathbf{U^{\prime}}} = \left[ {\begin{array}{*{20}c} 1 & 0 & 0 & 0 \\ 0 & 1 & 0 & 0 \\ 0 & 0 & 1 & 0 \\ \end{array} } \right]$$ and $${\mathbf{D^{\prime}}} = \left[ {\begin{array}{*{20}c} 3 & 0 & { - 5} \\ 0 & 3 & 2 \\ \end{array} } \right]$$ when we made selection. We estimated ${\mathbf{P}}$ and ${\mathbf{C}}$ by REML, and we denoted such estimates as ${\hat{\mathbf{P}}}$ and ${\hat{\mathbf{C}}}$. In addition, we use this dataset to compare the results of the maximized LPSI and CLPSI response (and correlation with the net genetic merit), when matrix ${\mathbf{C}}$ is known and when this matrix is estimated (${\hat{\mathbf{C}}}$).

### Real and simulated data availability

The real and simulated datasets are available in the *Application of a Genomic Selection Index to Real and Simulated Data* repository, at https://hdl.handle.net/11529/10199, where the folder of the real dataset is denoted as DATA_SET-3, whereas the folder of the simulated dataset is denoted as PSI_Phenotypes-05.

## Results

### Theoretical results

#### Distribution, expectation and variance of $S_{I}^{2}$ and $S_{{I_{C} }}^{2}$.

In Appendix B, we gave a brief description of the Fourier transform theory (Eqs. A6 to A8) used to find the distribution of $S_{I}^{2}$ and $S_{{I_{C} }}^{2}$. Based on the Springer (1979, Chapter 9) results, in Appendix C (Eqs. A9–A11), we present the mathematical process used to obtain the distribution of the $S_{I}^{2}$ and $S_{{I_{C} }}^{2}$ values, and we showed that the distribution of $S_{I}^{2}$ and $S_{{I_{C} }}^{2}$ is a Gamma distribution ($r$, $\lambda$), where $r = \frac{n - 2}{2}$ is the shape parameter and $\lambda = \frac{n - 1}{{2\sigma^{2} }}$ is the rate parameter (Stuart and Ord 1987). The distribution of $S_{I}^{2}$ and $S_{{I_{C} }}^{2}$ is essentially scaled Chi-squares ($r = \frac{n - 2}{2}$, a Chi-square with *n* − 2 degree of freedom and a scale of $\lambda = \frac{n - 1}{{2\sigma^{2} }}$). This is expected from their form as sums of squares of normally distributed data.

As shown in Appendix D (Eqs. A12–A15), the expectation and variance of $S_{I}^{2}$ and $S_{{I_{C} }}^{2}$ were the expectation of the Gamma distribution ($r$, $\lambda$). They are useful to obtain the expectation and variance of the estimator of the maximized LPSI (Eq. 12) and the maximized CLPSI (Eq. 13) selection responses. In $r$ and $\lambda$, $n$ is the size of the population in each selection cycle and $\sigma^{2}$ is a parameter that denotes the unknown and fixed variance of $I = {\mathbf{b^{\prime}y}}$ ($\sigma_{I}^{2} = {\mathbf{b^{\prime}Pb}}$) or the unknown and fixed variance of $I_{C} = {\mathbf{\beta^{\prime}y}}$ ($\sigma_{{I_{C} }}^{2} = {\mathbf{\beta^{\prime}P\beta }}$).

#### Expectation and variance of $\hat{R}_{\max } = k\sqrt {S_{I}^{2} }$ and $\hat{R}_{\max C} = k\sqrt {S_{{I_{C} }}^{2} }$.

In Appendix E (Eqs. A16 and A17), we give a brief description of the Delta method, which we used to determine the expectations and the variance of $\hat{R}_{\max } = k\sqrt {S_{I}^{2} }$ and $\hat{R}_{\max C} = k\sqrt {S_{{I_{C} }}^{2} }$. In this subsection, we present the expectations and variances only in terms of $\hat{R}_{\max }$; however, the results can be applied to any linear selection index with normal distribution.

Let $Y = k\sqrt {S^{2} } = \hat{R}$, where $k$ (the selection intensity) is a fixed constant, $\mu = E(S^{2} ) = \sigma_{I}^{2}$ and $Var(S^{2} ) = \frac{{2(\sigma_{I}^{2} )^{2} }}{n - 1}$ (Appendix D, Eqs. A14 and A15, respectively). According to the Delta method, the expectation, variance and standard deviation of $\hat{R}_{\max }$ are:

14 $$E(\hat{R}_{\max } ) \approx k\sigma_{I} - \frac{{k\sigma_{I} }}{4(n - 1)},$$

15 $$Var(\hat{R}_{\max } ) \approx \frac{{k^{2} \sigma_{I}^{2} }}{2(n - 1)},$$

16 $$SD(\hat{R}_{\max } ) \approx \frac{{k\sigma_{I} }}{{\sqrt {2(n - 1)} }},$$

respectively, where $\sigma_{I}^{{}} = \sqrt {{\mathbf{b^{\prime}Pb}}}$ and $\sigma_{I}^{2} = {\mathbf{b^{\prime}Pb}}$ are the unknown and fixed standard deviation and variance of $I = {\mathbf{b^{\prime}y}}$. The results of Eqs. (14) to (16) are the same for the CLPSI, changing $\sigma_{I}^{2} = {\mathbf{b^{\prime}Pb}}$ by $\sigma_{{I_{C} }}^{2} = {\mathbf{\beta^{\prime}P\beta }}$. In Eq. (14), the term $\frac{{k\sigma_{I} }}{4(n - 1)}$ is the bias of the estimator $\hat{R}_{\max }$ and the symbol “$\approx$” denotes an approximation. Equation (14) indicates that in the asymptotic context, $\hat{R}_{\max }$ is an unbiased estimator of ${R_{\max } = k\sqrt {{\mathbf{b^{\prime}Pb}}} }$, whereas Eq. (15) indicates that the variance of $\hat{R}_{\max }$ tends to zero when $n$ increases. That is, when the number of genotypes ($n$) increases in the training population, the particular realizations of $\hat{R}_{\max }$ will be concentrated around the $R_{\max }$ value. The same is true for the $\hat{R}_{\max C}$ values of the CLPSI and $R_{\max C} = k\sqrt {{\mathbf{\beta^{\prime}P\beta }}}$.

We can estimate Eqs. (14), (15) and (16) as

17 $$\hat{E}(\hat{R}_{\max } ) = kS_{I} - \frac{{kS_{I} }}{4(n - 1)},$$

18 $$\hat{V}ar(\hat{R}_{\max } ) = \frac{{k^{2} S_{I}^{2} }}{2(n - 1)},$$

19 $$S\hat{D}(\hat{R}_{\max } ) = \frac{{kS_{I} }}{{\sqrt {2(n - 1)} }},$$

respectively, where $S_{I}^{{}}$ and $S_{I}^{2}$ are the standard deviation and variance of the $\hat{I} = {\mathbf{\hat{b^{\prime}}y}}$ values in each selection cycle. The same is true for $S_{{I_{C} }}^{2}$ associated with the estimator of the maximized CLPSI selection response $\hat{R}_{\max C}$.

#### Desirable properties of the estimator of the maximized selection responses

An estimator should be unbiased, i.e., the expectation of the estimator should be equal to the parameter [$E(\hat{R}_{\max } ) = R_{\max }$], and the variance of the error of estimation [${\text{Var(}}R_{\max } - \hat{R}_{\max } {)}$] and the mean-squared error (MSE, i.e. $Var(\hat{R}_{\max } ) + [{\text{bias}}\hat{R}_{\max } ]^{2}$) should be minimum (Montgomery and Ruger 2003, Chapter 7). According to Eq. (14), $E(\hat{R}_{\max } ) = R_{\max }$ in the asymptotic context, and by Eq. (15), ${\text{Var(}}R_{\max } - \hat{R}_{\max } {\text{) = Var(}}\hat{R}_{\max } {)} \approx \frac{{k^{2} \sigma_{I}^{2} }}{2(n - 1)}$. In addition, because $\frac{{k\sigma_{I} }}{4(n - 1)}$ is the bias of $\hat{R}_{\max }$, $MSE = Var(\hat{R}_{\max } ) + [{\text{bias}}\hat{R}_{\max } ]^{2} \approx \frac{{k^{2} \sigma_{I}^{2} }}{2(n - 1)} + \frac{{k^{2} \sigma_{I}^{2} }}{{16(n - 1)^{2} }}$. We would expect that when the population size ($n$) is large, ${\text{Var(}}\hat{R}_{\max } {)}$ and MSE will be minimal. Eqs. (17) to (19) are useful to estimate ${\text{Var(}}\hat{R}_{\max } {)}$, $\frac{{k\sigma_{I} }}{4(n - 1)}$, and $MSE$.

#### A large-sample confidence interval for $E(\hat{R}_{\max } )$.

By the central limit theorem (Rencher 2002, Chapter 4), when the sample size $n$ is large (e.g., $n > 40$), the estimated expectation $\hat{E}(\hat{R}_{\max } )$ and the estimated standard deviation $S\hat{D}(\hat{R}_{\max } )$ allow constructing confidence intervals for $E(\hat{R}_{\max } )$. A confidence interval (CI) shows the likely range in which the $E(\hat{R}_{\max } )$ value would fall if the sampling exercise were to be repeated (Crawley 2015, Chapter 4). A large-sample confidence interval for $E(\hat{R}_{\max } )$ is

20 $$\hat{E}(\hat{R}_{\max } ) \pm Z_{\alpha /2} S\hat{D}(\hat{R}_{\max } ),$$

where $\hat{E}(\hat{R}_{\max } )$ and $S\hat{D}(\hat{R}_{\max } )$ were defined earlier, $Z_{\alpha /2}$ is the upper 100 $\alpha$/2 percentage point of the standard normal distribution, and $0 \le \alpha \le 1$ is the level of confidence. Thus, if for $E(\hat{R}_{\max } )$ we want to establish a $100(1 - \alpha )\% =$ 95% CI, in addition to $S\hat{D}(\hat{R}_{\max } )$, we need to obtain (from the standard normal distribution) the value of $Z_{\alpha /2}$ associated with $\frac{\alpha }{2} = \frac{0.05}{2} = 0.025$, i.e., $Z_{\alpha /2} = 1.96$. Equation (20) holds, regardless of the shape of the population distribution (Montgomery and Ruger 2003, Chapter 8).

#### Choice of sample size

By Eq. (20), the length or precision of the $100(1 - \alpha )\%$ CI for $E(\hat{R}_{\max } )$ is $2Z_{\alpha /2} S\hat{D}(\hat{R}_{\max } )$, whereas the error is $\varepsilon = \left| {\hat{E}(\hat{R}_{\max } ) - E(\hat{R}_{\max } )} \right|$, where $\left| \circ \right|$ denotes the absolute value of the difference $\hat{E}(\hat{R}_{\max } ) - E(\hat{R}_{\max } )$. In using $\hat{E}(\hat{R}_{\max } )$ to estimate $E(\hat{R}_{\max } )$, the error $\varepsilon$ is less than or equal to $S\hat{D}(\hat{R}_{\max } )$ with confidence $100(1 - \alpha )\%$. We can choose *n* so that we are $100(1 - \alpha )\%$ confident that the error in estimating $E(\hat{R}_{\max } )$ is less than a specified bound on the error $\varepsilon$ as follows

21 $$n = \left[ {\frac{{Z_{\alpha /2} S\hat{D}(\hat{R}_{\max } )}}{\varepsilon }} \right]^{2} .$$

If the right-hand side of Eq. (21) is not an integer, it must be rounded off. This will ensure that the level of confidence does not fall below $100(1 - \alpha )\%$ (Montgomery and Ruger 2003, Chapter 8). Equation (21) indicates that the lower the $\varepsilon$ value, the higher the $n$ size.

### Real data numerical results

#### Normality test for the estimated LPSI and CLPSI values

For the estimated LPSI values, the Shapiro–Wilk and Kolmogorov–Smirnov test values were 0.985 and 0.075, respectively, while for the estimated CLPSI values, those test values were 0.989 and 0.080, respectively. Thus, we assumed that the estimated indices values approach the normal distribution.

#### Histograms and quantile–quantile plots for the estimated LPSI and CLPSI values

With the estimated LPSI and CLPSI values, we constructed histograms (Fig. 1a, b) and quantile–quantile plots (Fig. 1c, d). The histograms of Fig. 1a, b of both indices do not show a strong negative or positive skew, while in Fig. 1c, d, the estimated LPSI and CLPSI values form a straight line in the quantile–quantile plots. Thus, the estimated LPSI and CLPS values approach the normal distribution.

#### Estimate of the maximized LPSI and CLPSI selection responses

For a selection intensity of 10% (*k* = 1.755), the estimate of the maximized LPSI response was 5.87, whereas the estimate of the maximized CLPSI selection response was 5.74. That is, the estimated selection responses of both indices were very similar. This means that the CLPSI constraint mainly affected the CLPSI expected genetic gains per trait.

#### Estimated bias, standard deviation and expectation of the estimator of the maximized LPSI and CLPSI selection responses

The bias of the estimator of the maximized LPSI and CLPSI selection responses was equal to 0.006. That is, the estimated bias was the same for both indices. In a similar manner, the standard deviation of the estimator of the maximized LPSI and CLPSI selection responses was 0.26, whereas the expectations of the estimator of the maximized LPSI and CLPSI selection responses were 5.86 and 5.73. These last two values were very similar to the estimated values of the maximized LPSI and CLPSI responses (5.87 and 5.74, respectively). The 95% confidence intervals for the $E(\hat{R}_{\max } )$ of the estimated LPSI and CLPSI selection responses were, respectively, (5.35, 6.37) and (5.22, 6.24).

#### Numerical results of the simulated data

For seven simulated selection cycles, in Table 1, we present the Shapiro–Wilk and Kolmogorov–Smirnov statistical test values, the estimated standard deviation, bias, the estimated mean-squared error ($MS\hat{E}$), the estimated maximized selection response ($\hat{R}_{\max }$), its estimated expectation [$E(\hat{R}_{\max } )$], and 95% confidence interval for the $E(\hat{R}_{\max } )$ of the LPSI and CLPSI, respectively.

**Table 1:** Shapiro–Wilk and Kolmogorov–Smirnov (SW and KS, respectively) statistical test values; estimated unconstrained and constrained linear phenotypic selection indices (LPSI and CLPSI, respectively) standard deviation (SD), bias, mean-squared error (MSE), maximized selection response ($\hat{R}_{\max }$ and $\hat{R}_{\max C}$), expectation [$\hat{E}(\hat{R}_{\max } )$ and $\hat{E}(\hat{R}_{\max C} )$], and 95% confidence interval (CI, *LCL *lower confidence limit, *UCL *upper confidence limit) for seven simulated selection cycles when the genotypic covariance matrix was estimated

	Statistical test		Estimated LPSI parameters					95% CI	
Cycle	SW	KS	SD	Bias	MSE	$\hat{R}_{\max }$	$\hat{E}(\hat{R}_{\max } )$	LCL	UCL
1	0.996	0.035	0.57	0.009	0.32	17.81	17.80	16.68	18.92
2	0.995	0.042	0.50	0.008	0.25	15.69	15.68	14.70	16.66
3	0.997	0.024	0.45	0.007	0.20	14.21	14.21	13.33	15.09
4	0.998	0.037	0.46	0.007	0.21	14.34	14.34	13.44	15.24
5	0.997	0.024	0.44	0.007	0.19	13.64	13.63	12.77	14.49
6	0.996	0.027	0.39	0.006	0.15	12.04	12.03	11.27	12.79
7	0.996	0.035	0.36	0.006	0.13	11.61	11.60	10.89	12.31
Average	0.997	0.032	0.46	0.007	0.21	14.19	14.18	13.30	15.07

	Statistical test		Estimated CLPSI parameters					95% CI	
Cycle	SW	KS	SD	Bias	MSE	$\hat{R}_{\max C}$	$\hat{E}(\hat{R}_{\max C} )$	LCL	UCL
1	0.998	0.024	0.50	0.008	0.25	15.79	15.78	14.80	16.76
2	0.996	0.032	0.47	0.008	0.22	14.98	14.97	14.05	15.89
3	0.998	0.024	0.42	0.007	0.18	13.58	13.57	12.75	14.39
4	0.998	0.038	0.39	0.006	0.15	12.36	12.36	11.60	13.12
5	0.996	0.025	0.40	0.006	0.16	12.80	12.79	12.01	13.57
6	0.995	0.025	0.36	0.006	0.13	11.23	11.23	10.52	11.94
7	0.995	0.031	0.36	0.006	0.13	11.23	11.23	10.52	11.94
Average	0.997	0.028	0.42	0.007	0.17	13.14	13.13	12.32	13.94

#### Normality test for the estimated LPSI and CLPSI values

The averages of the Shapiro–Wilk and Kolmogorov–Smirnov normality test values for the seven simulated selection cycles associated with the estimated LPSI values were 0.997 and 0.032, respectively, whereas those values associated with the estimated CLPSI values were 0.997 and 0.028 (Table 1), respectively; thus, we assumed that the estimated values of both indices approach the normal distribution.

#### Estimated standard deviation, bias and MSE of the estimator of the maximized LPSI and CLPSI selection responses

The averages of the estimated standard deviation of the estimator of the maximized LPSI and CLPSI selection responses were 0.46 and 0.42, respectively, whereas the average of the estimated bias for both indices was equal to 0.007. In addition, the averages of the estimated MSE of the estimator of the maximized LPSI and CLPSI selection responses were 0.21 and 0.17, respectively (Table 1). This means that the estimators of the maximized LPSI and CLPSI selection responses were good.

#### Estimates of the maximized LPSI and CLPSI selection responses, expectation and confidence intervals

For a selection intensity of 10% (*k* = 1.755), the averages of the estimates of the maximized LPSI and CLPSI selection response values were 14.19 and 13.14, respectively (Table 1). Thus, since the estimated responses of both indices were very similar, the CLPSI constraint mainly affected the CLPSI expected genetic gains per trait.

The averages of the estimated values of the expectations of the estimator of the maximized LPSI and CLPSI selection responses were 14.18 and 13.13. These last two values were very similar to the estimated values of the maximized LPSI and CLPSI responses (14.19 and 13.14, respectively). In addition, the averages of the estimated values of the 95% confidence intervals for the expectations of the estimator of the maximized LPSI and CLPSI selection responses were (13.30, 15.07) and (12.32, 13.94).

#### Estimator of the maximized LPSI and CLPSI selection responses using ${\mathbf{C}}$

For seven simulated selection cycles, in Table 2, we present the estimated LPSI and CLPSI standard deviation, bias, mean-squared error, maximized selection response, expectation, 95% confidence interval for $E(\hat{R}_{\max } )$ and $E(\hat{R}_{\max C} )$ and response upper bound when the genotypic covariance matrix ${\mathbf{C}}$ is known. When we compared those parameters with those obtained with ${\hat{\mathbf{C}}}$ (Table 1), we can see that the results were basically the same. That is, the estimated LPSI and CLPSI parameters were very similar when we used ${\hat{\mathbf{C}}}$ and ${\mathbf{C}}$. This means that the REML estimate ${\hat{\mathbf{C}}}$ is a good estimator of ${\mathbf{C}}$, at least for this simulated dataset. Finally, note that the average values of the upper boundary for $R$ ($k\sqrt {{\mathbf{w^{\prime}Cw}}}$) and $R_{C}$ ($k\sqrt {{\mathbf{\delta^{\prime}C\delta }}}$) presented in Table 2 were higher than estimated maximized LPSI and CLPSI selection responses for ${\hat{\mathbf{C}}}$ and ${\mathbf{C}}$, as we would expect.

**Table 2:** Estimates of the unconstrained and constrained linear phenotypic selection indices (LPSI and CLPSI, respectively) standard deviation (SD), bias, mean-squared error (MSE), maximized selection response ($\tilde{R}_{\max }$ and $\hat{R}_{\max C}$), expectation [$\tilde{E}(\tilde{R}_{\max } )$ and $\tilde{E}(\tilde{R}_{\max C} )$], 95% confidence interval (CI, *LCL *lower confidence limit, *UCL *upper confidence limit) for $E(\tilde{R}_{\max } )$ and response upper bound ($R_{\max }$ and $R_{\max C}$), for seven simulated selection cycles when the genotypic covariance matrix is known

	Estimated LPSI parameters when the genotypic covariance matrix is known							Upper bound
Cycle	SD	bias	MSE	$\tilde{R}_{\max }$	$\tilde{E}(\tilde{R}_{\max } )$	LCL	UCL	$R_{\max }$
1	0.556	0.009	0.309	17.559	17.550	16.469	18.648	19.63
2	0.480	0.008	0.231	15.179	15.172	14.238	16.121	17.56
3	0.451	0.007	0.204	14.261	14.254	13.376	15.146	16.49
4	0.437	0.007	0.191	13.797	13.790	12.941	14.653	16.32
5	0.435	0.007	0.189	13.742	13.735	12.889	14.594	15.99
6	0.392	0.006	0.154	12.387	12.381	11.619	13.156	14.69
7	0.409	0.006	0.168	12.935	12.928	12.132	13.737	14.90
Average	0.452	0.007	0.206	14.266	14.259	13.381	15.151	16.511

	Estimated CLPSI parameters when the genotypic covariance matrix is known							Upper bound
Cycle	SD	bias	MSE	$\tilde{R}_{\max C}$	$\tilde{E}(\tilde{R}_{\max C} )$	LCL	UCL	$R_{\max C}$
1	0.497	0.008	0.247	15.700	15.692	14.726	16.674	17.47
2	0.456	0.007	0.208	14.391	14.384	13.499	15.284	16.24
3	0.420	0.007	0.176	13.266	13.259	12.443	14.089	15.15
4	0.387	0.006	0.150	12.215	12.209	11.457	12.973	13.95
5	0.395	0.006	0.156	12.466	12.460	11.692	13.239	14.28
6	0.362	0.006	0.131	11.443	11.437	10.733	12.153	13.11
7	0.361	0.006	0.130	11.404	11.399	10.697	12.112	13.14
Average	0.411	0.007	0.171	12.984	12.977	12.178	13.789	14.763

#### Variance and confidence interval for the LPSI and CLPSI correlations using ${\mathbf{C}}$ and ${\hat{\mathbf{C}}}$

Using the known (${\mathbf{C}}$) and estimated (${\hat{\mathbf{C}}}$) genotypic covariance matrix, in Table 3, we present the estimated LPSI and CLPSI correlation coefficients when the genotypic covariance matrix is known ($\tilde{\rho }_{\max }$ and $\tilde{\rho }_{\max C}$) and estimated ($\hat{r}_{\max }$ and $\hat{r}_{\max C}$), standard deviation ($SD_{{\tilde{\rho }_{\max } }}$, $SD_{{\tilde{\rho }_{\max C} }}$, $SD_{{\hat{r}_{\max } }}$ and $SD_{{\hat{r}_{\max C} }}$), and 95% confidence intervals for the true unknown correlation ($\rho_{\max }$ and $\rho_{\max C}$) for seven simulated selection cycles. For both indices, the estimated parameters were very similar when we used ${\hat{\mathbf{C}}}$ and ${\mathbf{C}}$. This means that the REML estimate ${\hat{\mathbf{C}}}$ was a good estimator of ${\mathbf{C}}$, at least for this simulated dataset.

**Table 3:** Estimated unconstrained and constrained linear phenotypic selection indices (LPSI and CLPSI, respectively) correlation coefficients when the genotypic covariance matrix is known ($\tilde{\rho }_{\max }$ and $\tilde{\rho }_{\max C}$) and estimated ($\hat{r}_{\max }$ and $\hat{r}_{\max C}$); standard deviation ($SD_{{\tilde{\rho }_{\max } }}$, $SD_{{\tilde{\rho }_{\max C} }}$, $SD_{{\hat{r}_{\max } }}$ and $SD_{{\hat{r}_{\max C} }}$) and 95% confidence interval (CI, *LCL *lower confidence limit, *UCL *upper confidence limit) for the true unknown correlation ($\rho_{\max }$ and $\rho_{\max C}$) for seven simulated selection cycles

	LPSI correlation coefficient							
	Genotypic covariance matrix known				Estimated Genotypic covariance matrix			
Cycle	$\tilde{\rho }_{\max }$	$SD_{{\tilde{\rho }_{\max } }}$	LCL	UCL	$\hat{r}_{\max }$	$SD_{{\hat{r}_{\max } }}$	LCL	UCL
1	0.894	0.009	0.875	0.911	0.906	0.008	0.875	0.911
2	0.864	0.011	0.840	0.885	0.883	0.010	0.840	0.885
3	0.865	0.011	0.841	0.885	0.866	0.011	0.841	0.885
4	0.845	0.013	0.818	0.869	0.863	0.011	0.818	0.869
5	0.859	0.012	0.834	0.881	0.855	0.012	0.834	0.881
6	0.843	0.013	0.816	0.867	0.830	0.014	0.816	0.867
7	0.868	0.011	0.845	0.888	0.832	0.014	0.845	0.888
Average	0.863	0.011	0.839	0.884	0.862	0.011	0.839	0.884

	CLPSI correlation coefficient							
	Genotypic covariance matrix known				Estimated genotypic covariance matrix			
Cycle	$\tilde{\rho }_{\max C}$	$SD_{{\tilde{\rho }_{\max C} }}$	LCL	UCL	$\hat{r}_{\max C}$	$SD_{{\hat{r}_{\max C} }}$	LCL	UCL
1	0.800	0.016	0.766	0.829	0.803	0.016	0.769	0.832
2	0.819	0.015	0.788	0.846	0.842	0.013	0.815	0.866
3	0.804	0.016	0.771	0.833	0.827	0.014	0.797	0.853
4	0.748	0.020	0.707	0.785	0.744	0.020	0.702	0.781
5	0.779	0.018	0.742	0.812	0.803	0.016	0.769	0.832
6	0.779	0.018	0.742	0.811	0.775	0.018	0.738	0.808
7	0.765	0.019	0.727	0.800	0.805	0.016	0.772	0.834
Average	0.785	0.017	0.749	0.817	0.800	0.016	0.766	0.829

## Discussion

### The multivariate normality assumption

The study of quantitative traits (QTs) in plants and animals is based on the mean and variance of QT phenotypic values. Quantitative traits are phenotypic expressions of plant and animal characteristics that show continuous variability and are the result of many gene effects interacting among them and with the environment (Cerón-Rojas and Crossa 2018, Chapter 2). That is, QTs are the result of unobservable gene effects distributed across plant or animal genomes, which interact among themselves and with the environment to produce the observable characteristic plant and animal phenotypes. The traits that concern plant and animal breeders the most are QTs*.* They are particularly difficult to analyze because heritable variations of QTs are masked by larger nonheritable variations that make it difficult to determine the genotypic values of individual plants or animals (Smith 1936). However, since QTs usually have normal distribution, it is possible to apply normal distribution theory when analyzing this type of data.

In the context of plant and animal breeding, the most important distribution theory associated with the QTs is the multivariate normality distribution, which had been the basis for developing the LSI theory. Under the multivariate normal distribution assumption, means, variances and covariances completely describe the index and trait values. In addition, if the trait values are not correlated, they are independent; linear combinations of traits are normal; and even when the trait phenotypic values do not have normal distribution, this distribution serves as a useful approximation, especially in inferences involving sample mean vectors, which, by the central limit theorem, have multivariate normal distribution (Rencher 2002, Chapter 4). By this reasoning, a fundamental assumption in this work was that the trait values have multivariate normal distribution and that the net genetic merit and the index values have bivariate normal distribution. Under the latter assumption, the regression of the net genetic merit on any linear function of the phenotypic values is linear (Kempthorne and Nordskog 1959).

Based on the normality assumption of the estimated LPSI and CLPSI values, we obtained the expectation and variance of the estimator of the maximized LPSI and CLPSI selection responses. The histograms, quantile–quantile plots and the Shapiro–Wilk and Kolmogorov–Smirnov normality tests of the estimated LPSI and CLPSI values indicated that these values approached the normal distribution. Thus, our results were valid under the normality assumption of the estimated LPSI and CLPSI values.

### The expectation and variance of $S_{I}^{2}$ and $S_{{I_{C} }}^{2}$

The expectation and variance of $S_{I}^{2}$ and $S_{{I_{C} }}^{2}$ were the basis for obtaining the expectation and variance of the estimator of the maximized LPSI and CLPSI selection responses. According to Montgomery and Ruger (2003, Chapter 7), the expectations of $S_{I}^{2}$ and $S_{{I_{C} }}^{2}$ are unbiased. In addition, using the maximum likelihood estimator of the variance of the estimated LPSI and CLPSI values ($S_{I}^{2} = n^{ - 1} \sum\nolimits_{j = 1}^{n} {(\hat{I}_{j} - \hat{m})^{2} }$ and $S_{{I_{C} }}^{2} = n^{ - 1} \sum\nolimits_{j = 1}^{n} {(\hat{I}_{{C_{j} }} - \hat{\mu })^{2} }$, respectively), it can be shown that Eq. (A15) (Appendix D) can be written as $\frac{{2(\sigma_{I}^{2} )^{2} }}{n}$ (Stuart and Ord 1987, Chapter 10). These results were similar to our result and did not affect the expectation and variance of estimated maximized LPSI and CLPSI selection responses because, to obtain those expectation and variance, we assumed that $E(S_{I}^{2} ) = \sigma_{I}^{2}$.

Using the Delta method, Lynch and Walsh (1998, Appendix 1) showed that $\frac{{2(S_{I}^{2} )^{2} }}{n + 2}$ is an unbiased estimator the variance of $S_{I}^{2}$ (Eq. A15, Appendix D) when this is obtained as $\frac{{2(\sigma_{I}^{2} )^{2} }}{n}$. By the Lynch and Walsh (1998, Appendix 1) results, the bias of the expectation of the estimator of the maximized selection response can be written as $\frac{{k\sigma_{I} }}{4(n + 2)}$ and its estimates as $\frac{kS}{{4(n + 2)}}$. In a similar manner, the variance of the estimator of the maximized selection response can be written as $\frac{{k^{2} \sigma_{I}^{2} }}{2(n + 2)}$ and its estimates as $\frac{{k^{2} S_{{}}^{2} }}{2(n + 2)}$. We would expect that the difference between the results we obtained with our equations and those that are possible to obtain with the Lynch and Walsh (1998, Appendix 1) results would be minimal.

Let $MSE_{1}$ be the mean-squared error of the estimator of the variance of the selection response when we use Eq. (A15, Appendix D), and let $MSE_{2}$ be the mean-squared error of the estimator of the variance of the selection response when we use $\frac{{2(S_{I}^{2} )^{2} }}{n + 2}$ to estimate $Var(\hat{R})$ (Eq. 15). Montgomery and Ruger (2003, Chapter 7) have indicated that a good criterion for comparing the relative efficiency of two different estimators is the ratio $\frac{{MSE_{1} }}{{MSE_{2} }}$. In the present case, this ratio is equal to $\frac{{MSE_{1} }}{{MSE_{2} }} = \frac{{(n + 2)^{2} [8(n - 1) + 1]}}{{(n - 1)^{2} [8(n + 2) + 1]}}$, which is independent of $S_{I}^{2}$, and when $n$ is large, it is close to 1.0, as we would expect. Thus, we would expect that both approaches would be similar.

### The standard deviation of $S_{I}^{2}$ and $S_{{I_{C} }}^{2}$.

Due to Jensen’s inequality, $E(S_{I} ) = E[(S_{I}^{2} )^{1/2} ] < [E(S_{I}^{2} )]^{1/2} = \sigma_{I}$ (Patel and Read 1996, Chapter 5). This means that the standard deviation of the variance of the estimated values of the LPSI and CLPSI ($S_{I}$ and $S_{{I_{C} }}$, respectively) subestimates $\sigma_{I} = \sqrt {{\mathbf{b^{\prime}Pb}}}$ and $\sigma_{{I_{C} }} = \sqrt {{\mathbf{\beta^{\prime}P\beta }}}$.

An unbiased estimator of $\sigma_{I}$ ($\sigma_{{I_{C} }}$) is $S_{I} /c(n)$[i.e., $E(S_{I} ) = c(n)\sigma_{I}$], where $c(n) = \sqrt {\frac{2}{n - 1}} \frac{\Gamma (n/2)}{{\Gamma \left( {\frac{n - 1}{2}} \right)}} \approx 1 - \frac{1}{4n} - \frac{7}{{32n^{2} }} - \frac{19}{{128n^{3} }}$ is a factor of correction (Johnson et al. 1994, Chapter 13; Montgomery and Ruger 2003, Chapter 7). However, when we used $c(n)$ to correct $S_{I}$ (data no presented), we did not find that $c(n)$ affects the expectation and variance of the estimated selection response. Johnson et al. (1994, Chapter 13) found that, in practice, $c(n)$ only affects $S_{I}$ when $n \le 10$. Thus, when $n = 247$ (real data) or $n = 500$ (simulated data), the results shall not be affected by $c(n)$.

Note that $c(n)\sigma_{I}$ is the expectation of a Nakagami-m distribution (Ramos et al. 2015). Patel and Read (1996, Chapter 5) indicated that such result is valid only when $E(S_{I} )$ is obtained with respect to the origin of the distribution of $S_{I}$, but when this expectation is obtained with respect to the average value of $S_{I}$, there is no concise expression for $E(S_{I} )$. These authors presented equations for the expectation and variance of $S_{I}$ that are very similar to those presented in Eqs. (14) and (15) of this work. That is, the Patel and Read (1996) results were in agreement with our results.

### The constrained LPSI (CLPSI)

The CLPSI solved the LPSI equations subject to the restriction that the covariance between the CLPSI and some linear combinations of the genotypes involved be equal to a vector of predetermined proportional gains (or constraints) imposed by the breeder. These constraints are similar to the null restriction imposed by the restricted LPSI (RLPSI), which imposes restrictions equal to zero on the expected genetic advances of some traits, while the expected genetic advances of other traits increased (or decreased) without imposing any restrictions. The RLPSI solves the usual LPSI equations subject to the restriction that the covariance between the LPSI and some linear functions of the genotypes involved be equal to zero, thus preventing selection on the index from causing any genetic change in the expected genetic advance of the restricted traits (Cunningham et al. 1970). Although both constraints are similar, their effects on the maximized selection response and expected genetic gain per trait, and coefficient of correlation, are different.

The RLPSI uses a projector matrix to project the LPSI vector of coefficients into a space smaller than the original space of the LPSI vector of coefficients. The reduction of the space into which the RLPSI matrix projects the LPSI vector of coefficients is equal to the number of zeros that appears in the expected genetic gain per trait, and the selection response and correlation coefficient decrease as the number of restrictions increases (Cerón-Rojas and Crossa, 2018, Chapter 3). Nevertheless, the CLPSI constraints affect only the expected genetic gain pert trait, not the maximized CLPSI selection response (Cerón-Rojas and Crossa 2019). In addition, the maximized CLPSI correlation coefficient is only affected when the number of constraints is equal to or higher than three, but even in this last case, such affectation could be not significant, as we saw in this work. Thus, the CLPSI is a good predictor of the net genetic merit and breeder could use it with confidence.

### The estimated LPSI and CLPSI parameters when the genotypic covariance matrix is known and estimated

While the sampling properties of the estimator of the phenotypic covariance matrix are well known (Rencher and Schaalje 2008), the sampling properties of the estimator of the genotypic covariance matrix are not well known. By this reason, in this work, we estimated and compared the LPSI and CLPSI parameters when the genotypic covariance matrix is known and estimated. The results indicated that the differences were not significant; thus, when the phenotypic and genotypic covariance matrices are estimated by REML, breeder could use LPSI and CLPSI with confidence.

### Other LSIs associated with the LPSI and CLPSI

The LPSI and the CLPSI are optimal LSIs when the phenotypic (${\mathbf{P}}$) and the genotypic (${\mathbf{C}}$) covariance matrices are known. In practice, however, it is necessary to estimate such matrices. When the estimator of the phenotypic covariance matrix (${\hat{\mathbf{P}}}$) is not positive definite (all eigenvalues positive) or the estimator of the genotypic covariance matrices (${\hat{\mathbf{C}}}$) is not positive semidefinite (no negative eigenvalues), the estimator of the LPSI and CLPSI vector of coefficients could be biased when the sample size is low. For this reason, Williams (1962b) proposed using the base linear phenotypic selection index ($I_{B} = {\mathbf{w^{\prime}y}}$) which could be a better predictor of $H = {\mathbf{w^{\prime}g}}$ than the *estimated* LPSI $\hat{I} = {\mathbf{\hat{b^{\prime}}y}}$ if indeed the vector of economic values ${\mathbf{w}}$ is known. If vector ${\mathbf{w}}$ values is known, then $I_{B}$ has certain advantages because of its simplicity and its freedom from parameter estimation errors. Williams (1962b) pointed out that the $I_{B}$ is superior to $\hat{I}$ unless a large amount of data is available for estimating ${\mathbf{P}}$ and ${\mathbf{C}}$; however, the availability of accurate and fast algorithms for estimating ${\mathbf{P}}$ and ${\mathbf{C}}$ by REML, such as those implemented in *RIndSel* (Cerón-Rojas and Crossa 2018, Chapter 11), makes $\hat{I}$ a good option to make selection. RIndSel (R software to analyze Selection Indices) is a graphical unit interface that uses selection index theory to select individual candidates as parents for the next selection cycle in the phenotypic and genomic selection context.

There are some problems associated with $I_{B}$. For example, what is its selection response when no data are available for estimating ${\mathbf{P}}$ and ${\mathbf{C}}$? $I_{B}$ is a better selection index than the LPSI only if the correlation between $I_{B}$ and the net genetic merit is higher than that between the LPSI and the net genetic merit (Hazel 1943). But if estimations of ${\mathbf{P}}$ and ${\mathbf{C}}$ are not available, how can we obtain the correlation between the base index and the net genetic merit? Williams (1962a) pointed out that the correlation between $I_{B}$ and $H$ can be written as $\rho_{B} = \sqrt {\frac{{{\mathbf{w^{\prime}Cw}}}}{{{\mathbf{w^{\prime}Pw}}}}}$ and indicated that the ratio $\rho_{B} /\rho$ ($\rho$ is the correlation between the LPSI and $H$; see Eqs. 3 and 4b) can be used to compare LPSI efficiency *vs.* $I_{B}$ efficiency; however, in the latter case, we at least need to know the estimates of ${\mathbf{P}}$ and ${\mathbf{C}}$, *i.e*., ${\hat{\mathbf{P}}}$ and ${\hat{\mathbf{C}}}$. For this reason, we think that breeders should use the LPSI when the population size is sufficiently large.

An index similar to the CLPSI described in this work is the desired gains linear phenotypic selection index (Pesek and Baker 1969). The most important aspect of this last index is that it does not require economic weights. The main problem of this index is that it does not maximize the correlation between $I$ and $H$ ($\rho$) nor the selection response because the covariance between $I$ and $H$ ($Cov(H,I) = {\mathbf{w^{\prime}Cb}}$) is not defined, given that ${\mathbf{w^{\prime}Cb}}$ requires the economic weight vector ${\mathbf{w^{\prime}}}$ and that index does not use economic weights (Itoh and Yamada 1986, 1988). Another problem with this index is that it is not associated with $H$; then, it is not a predictor of $H$ and the $\rho$ and the selection response could not be maximum. For this reason, we think that breeders should use the CLPSI described in this work when making selection.

## Conclusions

We described a method to obtain the expectation and variance of the estimator of the maximized selection response for unconstrained and constrained linear phenotypic selection indices. The estimator of the maximized selection response was the square root of the variance of the estimated LSI values multiplied by the selection intensity. The expectation and variance allow the breeder to construct confidence intervals and determine the appropriate sample size to complete the analysis of a selection process. We validated the theoretical results in the phenotypic selection context using real and simulated datasets. We concluded that our results are valid for any LSI with normal distribution and that the method described in this work is useful for finding the expectation and variance of the estimator of any LSI response in the phenotypic or genomic selection context.

## Appendix A

### Upper boundary for the maximized LPSI and CLPSI selection response

By the Cauchy–Schwarz inequality (Sorensen and Gianola 2002, Chapter 2), the relationship among the variance of $H = {\mathbf{w^{\prime}g}}$ and $I = {\mathbf{b^{\prime}y}}$ (${\mathbf{w^{\prime}Cw}}$ and ${\mathbf{b^{\prime}Pb}}$, respectively), and the covariance (${\mathbf{w^{\prime}Cb}}$), is $({\mathbf{w^{\prime}Cb}})^{2} \le ({\mathbf{w^{\prime}Cw}})({\mathbf{b^{\prime}Pb}})$. But because the LPSI vector of coefficients is ${\mathbf{b}} = {\mathbf{P}}^{ - 1} {\mathbf{Cw}}$, that relationship can be written as

A1 $${\mathbf{w^{\prime}CP}}^{ - 1} {\mathbf{Cw}} \le {\mathbf{w^{\prime}Cw}}.$$

The maximized LPSI selection response is $R_{\max } = k\sqrt {{\mathbf{b^{\prime}Pb}}} = k\sqrt {{\mathbf{w^{\prime}CP}}^{ - 1} {\mathbf{Cw}}}$; thus, the upper boundary for $R_{\max }$ is $k\sqrt {{\mathbf{w^{\prime}Cw}}}$, i.e.,

A2 $$R_{\max } = k\sqrt {{\mathbf{w^{\prime}CP}}^{ - 1} {\mathbf{Cw}}} \le k\sqrt {{\mathbf{w^{\prime}Cw}}} .$$

Equation (A2) indicates that if ${\mathbf{P}} = {\mathbf{C}}$, then ${\mathbf{b}} = {\mathbf{w}}$ and $R_{\max } = k\sqrt {{\mathbf{w^{\prime}Cw}}}$. This is the maximum possible value of $R_{\max }$. Cerón-Rojas and Crossa (2019) showed that the upper boundary for the maximized CLPSI selection response is

A3 $$k\sqrt {{\mathbf{\delta^{\prime}C\delta }}} ,$$

where $${{\varvec{\updelta}}} = {\mathbf{K}}_{T} {\mathbf{w}}$$, $${\mathbf{K}}_{T} = [{\mathbf{I}}_{t} - {\mathbf{Q}}_{T} ]$$ and $${\mathbf{Q}}_{T} = {\mathbf{UD}}({\mathbf{D^{\prime}U^{\prime}CUD}})^{ - 1} {\mathbf{D^{\prime}U^{\prime}C}}$$.

### Variance and confidence intervals for $\rho_{\max }$

As $H = {\mathbf{w^{\prime}g}}$ and $I = {\mathbf{b^{\prime}y}}$ have bivariate normal distribution, the standard deviation of the variance of $\rho_{\max }$ is

A4 $$\frac{{(1 - \rho_{\max }^{2} )}}{\sqrt n },$$

while an approximated 100(1 − $\alpha$)% confidence interval for $\rho_{\max }$ is

A5 $$\tanh \left( {v - \frac{{Z_{\alpha /2} }}{{\sqrt {n - 3} }}} \right) \le \rho_{\max } \le \tanh \left( {v + \frac{{Z_{\alpha /2} }}{{\sqrt {n - 3} }}} \right),$$

where $\tanh ( \circ )$ is the hyperbolic tangent function and $v = \tanh^{ - 1} (\hat{r}_{\max } )$ its inverse, whereas $\hat{r}_{\max }$ is an estimate of $\rho_{\max }$, $Z_{\alpha /2}$ is the upper 100 $\alpha$/2 percentage point of the standard normal distribution, and $0 \le \alpha \le 1$ is the level of confidence (Rencher and Schaalje 2008, Chapter 10). Results of Eqs. (A4) and (A5) are also valid for the CLPSI.

## Appendix B

### Fourier transform ($F_{t} [f_{X} (x)]$) of $f_{X} (x)$

The basis for analyzing distributions of sums of continuous random variables that take on both positive and negative values is the Fourier transform, which allows deriving the probability density function of their sums. In this appendix, we give a brief review of the Fourier transform theory.

Let $f_{X} (x)$, $- \infty < x < \infty$, a single-valued real function such that the integral

A6 $$\int_{ - \infty }^{\infty } {\left| {f_{X} (x)} \right|e^{itx} dx}$$

converges for some real value of $t$, where $i = \sqrt { - 1}$ and $\left| \circ \right|$ denote the absolute value; then, $f_{X} (x)$ is said to be Fourier transformable, and

A7 $$F_{t} [f_{X} (x)] = \int_{ - \infty }^{\infty } {e^{itx} f_{X} (x)dx}$$

is the Fourier transform of $f_{X} (x)$ (Springer 1979, Chapter 2). Equation (A7) is also called the characteristic function of the random variable $X$ and can be denoted as $\phi_{X} (t) = E(e^{itX} )$. This is the expectation of a complex function, and since $\left| {e^{itx} } \right| = \left| {\cos tX + i\sin tX} \right| = 1$, Equation (A7) always exists. Furthermore, when $t = 0$, $\phi_{X} (0) = 1$ and $\left| {\phi_{X} (t)} \right| \le 1$ (Soong 2004, Chapter 4).

For $F_{t} [f_{X} (x)]$, there is a corresponding inverse transform, which can be written as

A8 $$f_{X} (x) = \frac{1}{2\pi }\int_{ - \infty }^{\infty } {e^{ - itx} F_{t} [f_{X} (x)]dt} .$$

Equation (A8) shows that knowledge of the Fourier transform, or characteristic function (Eq. A7) specifies the distribution of *X*. Furthermore, $f_{X} (x)$ is uniquely determined from Eq. (A8); that is, no two distinct density functions can have the same characteristic function (Springer 1979, Chapter 2; Soong 2004, Chapter 4).

## Appendix C

### Distribution of $S_{I}^{2}$ and $S_{{I_{C} }}^{2}$

In this appendix, under the assumption that the estimated LPSI and CLPSI values are normally distributed, we used the Fourier transform to obtain the distribution of $S_{I}^{2}$ and $S_{{I_{C} }}^{2}$ (Eqs. 10 and 11, respectively).

Suppose that $\hat{I}_{1}$,$\hat{I}_{2}$ …, $\hat{I}_{n}$ is a random sample of size $n$ of estimated index values (LPSI or CLPSI) and that $\sum\nolimits_{j = 1}^{n} {\hat{I}_{j} } = 0$. Let $S^{2} = \frac{1}{n - 1}\sum\nolimits_{j = 1}^{n} {\hat{I}_{j}^{2} }$ be an estimator of the variance ($\sigma^{2}$) of the index (in the LPSI context $\sigma^{2} = {\mathbf{b^{\prime}Pb}}$, whereas in the CLPSI context, $\sigma^{2} = {\mathbf{\beta^{\prime}P\beta }}$). That is, we are assuming that in each selection cycle, the estimated index values are a random sample of the distribution of all possible estimated index values.

To simplify notation, let $\hat{I}_{1} = X_{1}$, $\hat{I}_{2} = X_{2}$ …, $\hat{I}_{n} = X_{n}$ and suppose that we obtain the sample $n$ of estimated index values from the normal distribution $f_{X} (x) = \frac{1}{{\sigma \sqrt {2\pi } }}\exp \left\{ { - \frac{{x^{2} }}{{2\sigma^{2} }}} \right\}$, $- \infty < x < \infty$. Let $N = n - 1$ ($n =$ number of index values in each selection cycle) and $U = \sum\nolimits_{i = 1}^{n} {X_{i}^{2} } = NS^{2}$, subject to $\sum\nolimits_{j = 1}^{n} {X_{i} } = 0$, where $S^{2} = \frac{1}{n - 1}\sum\nolimits_{j = 1}^{n} {X_{j}^{2} }$ is an estimator of the variance ($\sigma^{2}$) of the LSI. By Eq. (A7), the Fourier transform of $g_{U} (u)$ is

A9 $$F_{t} [g_{U} (u)] = \left[ {\int_{ - \infty }^{\infty } {\frac{1}{{\sigma \sqrt {2\pi } }}} \exp \left\{ { - \frac{{x^{2} }}{{2\sigma^{2} }} + itx^{2} } \right\}dx} \right]^{N - 1} = [1 - 2it]^{{ - \frac{N - 1}{2}}}$$

which is the characteristic function of a Chi-square distribution with $N$-1 degrees of freedom (or $n - 2$ degrees of freedom because $N = n - 1$). In addition, by Eq. (A8)

A10 $$g_{U} (u) = \frac{1}{2\pi }\int_{ - \infty }^{\infty } {\frac{{e^{ - itu} }}{{(1 - 2it)^{(N - 1)/2} }}dt} = \frac{{u^{(N - 3)/2} e^{{ - (u/2\sigma^{2} )}} }}{{(2\sigma^{2} )^{(N - 1)/2} \Gamma [(N - 1)/2]}}$$

is the inverse transform of the Fourier transform of Eq. (A9) (Springer 1979, Chapter 9).

Let $h_{{S^{2} }} (s^{2} )$ be the density function of $S^{2}$. It follows from Eq. (A10) and the relationships $U = \sum\limits_{i = 1}^{n} {X_{i}^{2} } = NS^{2}$ and $du = NdS^{2}$ (where $du$ and $dS^{2}$ are differentials) that

A11 $$h_{{S^{2} }} (s^{2} ) = \left( {\frac{N}{{2\sigma^{2} }}} \right)^{(N - 1)/2} \frac{{(s^{2} )^{(N - 3)/2} e^{{ - (Ns^{2} /2\sigma^{2} )}} }}{\Gamma [(N - 1)/2]}$$

is the distribution function of $S^{2}$ (Springer 1979, Chapter 9), where for $r = \frac{N - 1}{2}$,$\Gamma (r) = \int_{0}^{\infty } {e^{ - z} z^{r - 1} dz}$ is the Gamma function (Stuart and Ord 1987, Chapter 5). Let $V = S^{2}$, $\lambda = \frac{N}{{2\sigma^{2} }}$ and $r = \frac{N - 1}{2}$; then, Eq. (A11) can be written as

A12 $$h_{V} (v) = \frac{{\lambda^{r} v^{r - 1} e^{ - \lambda v} }}{\Gamma (r)},$$

which is a Gamma distribution ($r$, $\lambda$), where for $0 < v < \infty$, $r > 0$ is the shape parameter, $\lambda$ is the rate parameter and $\Gamma (r) = \int_{0}^{\infty } {e^{ - z} z^{r - 1} dz}$ is defined earlier.

## Appendix D

### The expectation and variance of $S_{I}^{2}$ and $S_{{I_{C} }}^{2}$

The characteristic function of Eq. (A12) is $\phi (t) = \left( {1 - \frac{it}{\lambda }} \right)^{ - r}$, and the expectation and variance of $V = S^{2}$ are

A13 $$\frac{r}{\lambda }\quad {\text{and}}\quad \frac{r}{{\lambda^{2} }},$$

respectively (Stuart and Ord 1987, Chapter 5).

In the LPSI context, let $\sigma^{2} = {\mathbf{b^{\prime}Pb}}$ be the unknown variance of the LPSI; then, by Eq. (A13), the expectation and variance of $S^{2}$ are

A14 $$E(S^{2} ) = \frac{r}{\lambda } = \frac{n - 2}{{n - 1}}\sigma^{2} \approx \sigma^{2}$$

and

A15 $$Var(S^{2} ) = \frac{r}{{\lambda^{2} }} = \frac{2(n - 2)}{{(n - 1)^{2} }}(\sigma^{2} )^{2} \approx \frac{{2(\sigma^{2} )^{2} }}{n - 1}$$

, respectively. In Eqs. (A14) and (A15), the symbol “$\approx$” indicates an approximation. Equation (A14) indicates that $S^{2}$ is an asymptotic unbiased estimator of $\sigma^{2} = {\mathbf{b^{\prime}Pb}}$, whereas Eq. (A15) indicates that $Var(S^{2} )$ tends to zero when $n$ increases. Equations (A14) and (A15) are valid for CLPSI.

## Appendix E

### The Delta method

We determined the expectation and the variance of the estimator of the maximized LPSI and CLPS the selection responses using the Delta method (Lynch and Walsh 1998, Appendix 1; Sorensen and Gianola 2002, Chapter 2; Cerón-Rojas and Sahagún-Castellanos 2007, Appendix B). To find the expectation and variance of the estimator of the of the maximized LPSI and CLPSI selection response, we need to expand the function $Y = f(X)$ as a Taylor series around the expectation of the estimator of the maximized LPSI and CLPS selection response and then find the expectation and variance of the expansion of $Y = f(X)$. The first and second derivatives of the function are sufficient to obtain results that are very close to the expected results.

Suppose that $X$ is a random variable with mean $\mu$($E(X) = \mu$) and that $Y = f(X)$ is a function of $X$; then, approximations of the expectation and variance of $Y$ are obtained as

A16 $$E(Y) \approx f(\mu ) + \frac{1}{2}\frac{{d^{2} }}{{dX^{2} }}f(X)\left| {_{X = \mu } } \right.Var(X)$$

and

A17 $$Var(Y) \approx \left[ {\frac{d}{dX}f(X)\left| {_{X = \mu } } \right.} \right]^{2} Var(X),$$

respectively, where $\frac{d}{dX}f(X)\left| {_{X = \mu } } \right.$ and $\frac{1}{2}\frac{{d^{2} }}{{dX^{2} }}f(X)\left| {_{X = \mu } } \right.$ are the first and second derivatives of $f(X)$ with respect to $X$ evaluated at $\mu$, and $Var(X)$ is the variance of $X$.
